# A Rare Presentation of Lemierre’s Syndrome Caused by an Atypical Occurrence of Eikenella corrodens

**DOI:** 10.7759/cureus.78526

**Published:** 2025-02-04

**Authors:** Sadiq A Shah, Ibrahim Kazi, Adnan Younus, Noman Salih, Kazi N Khan

**Affiliations:** 1 Internal Medicine, Hayatabad Medical Complex Peshawar, Peshawar, PAK; 2 School of Medicine, University College Cork, Cork, IRL; 3 Internal Medicine, TidalHealth Peninsula Regional, Salisbury, USA; 4 Nephrology, Georgetown University, Washington, D.C., USA

**Keywords:** cerebral embolization, focal seizures, internal jugular vein, lemierre’s syndrome, septic thrombophlebitis, tooth extraction

## Abstract

Lemierre's syndrome is characterized by a septic inflammatory condition involving internal jugular veins and is frequently caused by an oropharyngeal infection. While it usually results in pulmonary embolization, this case demonstrates a rare but significant cerebral embolization. The case involves an 18-year-old woman who experienced left-sided neck swelling and difficulty swallowing five days after a tooth extraction, an uncommon trigger. Her symptoms progressed to a headache, localized seizures, and mental state changes. Upon hospital admission, she was febrile, had noticeable neck swelling, and a reduced Glasgow Coma Scale score. CT scans of her neck and her brain revealed an occurrence of septic emboli, indicating a septic thrombophlebitis that affected the internal jugular vein. As surgical intervention is rarely required in such cases, her treatment involved antibiotics and symptom management.

## Introduction

Lemierre's syndrome, frequently referred to as jugular venous suppurative thrombophlebitis, is an uncommon but dangerous illness that was initially identified by Andre Lemierre in 1936. It involves inflammation and clot formation in the internal jugular vein, often accompanied by an infection known as septic thrombophlebitis [1,2].

Although more common before the widespread use of antibiotics, Lemierre’s syndrome is now estimated to occur in approximately 3.6 million people annually [3]. Typically, it begins with a sore throat but can also be triggered by dental infections, tonsillitis, or other infections [4]. The infection starts in the throat and spreads to the neck vessels, leading to inflammation and clotting in the jugular vein and sometimes the carotid artery [3]. Although it usually affects the lungs, causing abscesses or empyema, this report focuses on the rarer but significant spread to the brain [5]. It can also impact the joints, liver, muscles, and covering of the heart i.e. pericardium, and skin [6].

Although additional species, such as *Enterobacteriaceae, Eikenella corrodens, Bacteroides, streptococci, *and* Staphylococcus aureus*, can be involved,* Fusobacterium necrophorum* remains the most commonly implicated pathogen. *Eikenella corrodens*, which can penetrate deep tissues, form biofilms, and contribute to polymicrobial infections, often alongside other anaerobes, may also play a pathogenic role in Lemierre's syndrome. It has been linked to lung infections, osteomyelitis, and septic thrombophlebitis [7]. Treatment typically involves specific antibiotics, and in certain cases, surgery or blood thinners may be necessary [8]. Physicians often prescribe antibiotics such as piperacillin-tazobactam, ampicillin-sulbactam, or meropenem [9]. In rare instances, jugular vein surgery may be required [10].

## Case presentation

An 18-year-old female patient, without notable medical history, presented to our facility reporting a five-day duration of fever, dysphagia, and neck swelling. The onset of fever was sudden, characterized by high-temperature spikes occurring intermittently. Dysphagia manifested approximately one day after the fever's onset, coinciding with the emergence of noticeable neck swelling. The patient disclosed undergoing a dental extraction one week prior to the commencement of these symptoms. During her initial evaluation, she arrived with a temperature of 104 Fahrenheit (40°C), a blood pressure measurement of 110/80 mm of mercury, a heart rate of 86 beats every minute, and a rate of breathing of 14 breaths per minute. Her oxygen saturation level was normal at 95%. Swelling and tenderness were observed on the left side of her neck. The swelling was non-tender, non-fluctuant, non-erythematous, and not fixed. Her tonsils were enlarged to a grade of 2+. The throat appeared erythematous without exudates, and her mucous membranes were somewhat dry. Lymphadenopathy was noted in the anterior cervical lymph nodes. Chest auscultation revealed clear lungs without any tenderness to percussion. A skin examination did not reveal any notable findings.

Laboratory tests indicated neutrophilic leukocytosis and increased inflammatory markers, notably an increase in erythrocyte sedimentation rate (ESR) as well as C-reactive protein (CRP). Initial tests, including the rapid streptococcal antigen test and the monospot test, were both negative. In addition, key baseline measurements from renal function tests and liver function tests, as well as electrolyte levels, were all consistently within the normal ranges. Some of the laboratory investigations are detailed in Table 1.

**Table 1:** Relevant laboratory investigations HB: hemoglobin, CRP: C-reactive protein, MCV: mean cell volume, ESR: erythrocyte sedimentation rate, tag: triacyl glycerol, WBC: white blood cells, LDL: low-density lipoprotein

Test	Patient's values	Reference ranges
Hb (g/dl)	11.9	11.55 - 17.0
Platelets (/µl)	184000	145000 - 455000
Wbcs (/µl)	13500	4000 - 11000
MCV (fL)	85.5	80-100
Ferritin (ng/mL)	345	12 - 150
ESR (mm/hr)	65	≤20
TAG (mg/dl)	90	<150
CRP (mg/dL)	20	<1.0
LDL cholesterol (mg/dl)	55	<100

A neck ultrasound was performed under the suspicion of a cervical abscess; however, it revealed thrombosis of the left internal jugular vein, which was associated with soft tissue edema. A throat swab for culture and sensitivity unexpectedly isolated *Eikenella corrodens* rather than the more typical* Fusobacterium necrophorum* associated with Lemierre’s syndrome. The organism demonstrated sensitivity to cephalosporins, including ceftazidime and ceftriaxone, as well as fluoroquinolones, such as ciprofloxacin, while exhibiting resistance to ampicillin and tetracyclines.

Lemierre's syndrome was diagnosed based on its clinical manifestation, imaging, and laboratory results. Ceftazidime was prescribed as part of the patient's targeted antibiotic treatment. Despite the associated controversy, oral anticoagulation therapy was initiated to mitigate thrombotic events.

**Figure 1:**
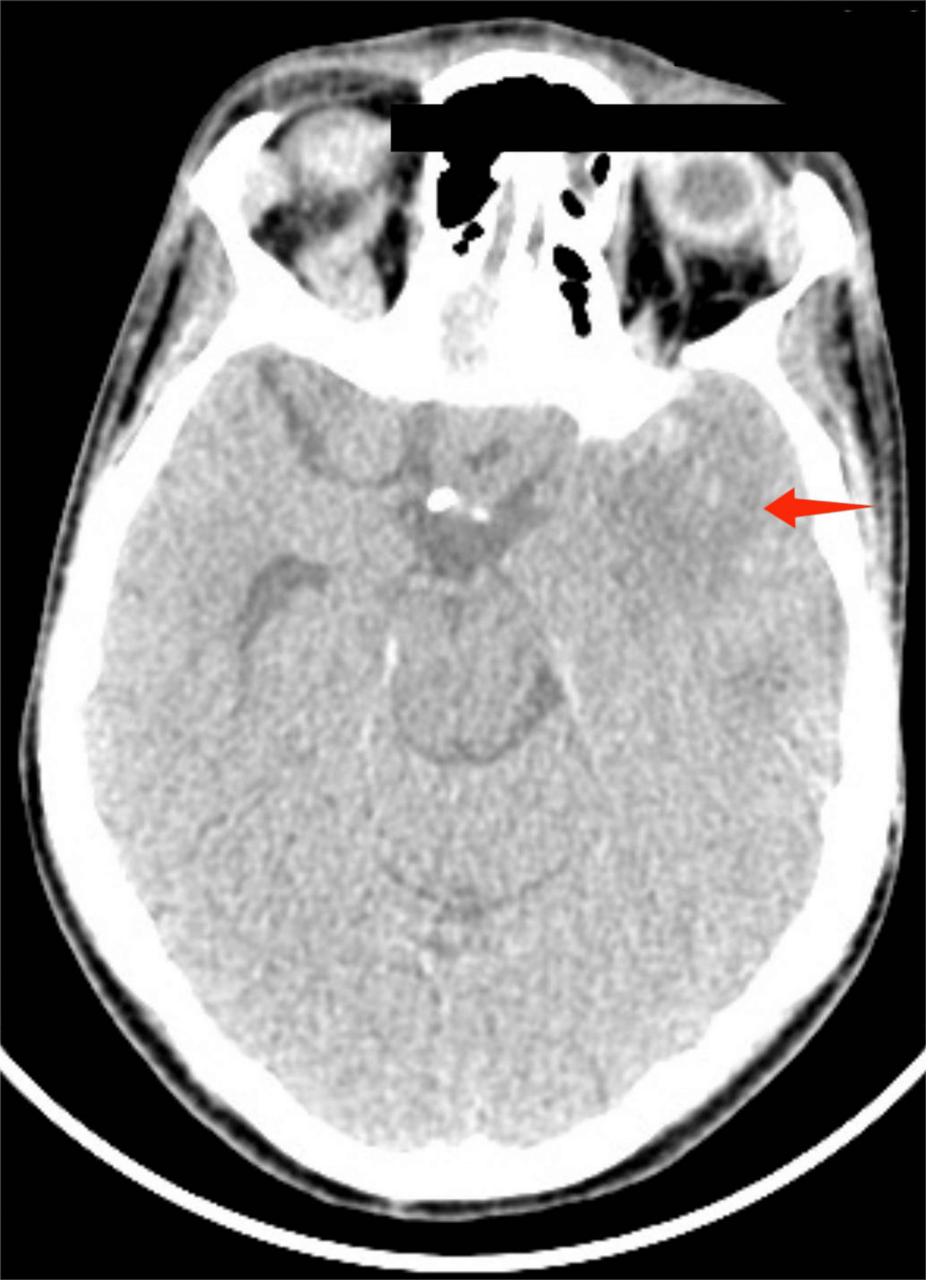
CT brain without contrast-axial view; the red arrow shows the dense left middle cerebral artery thrombus

**Figure 2:**
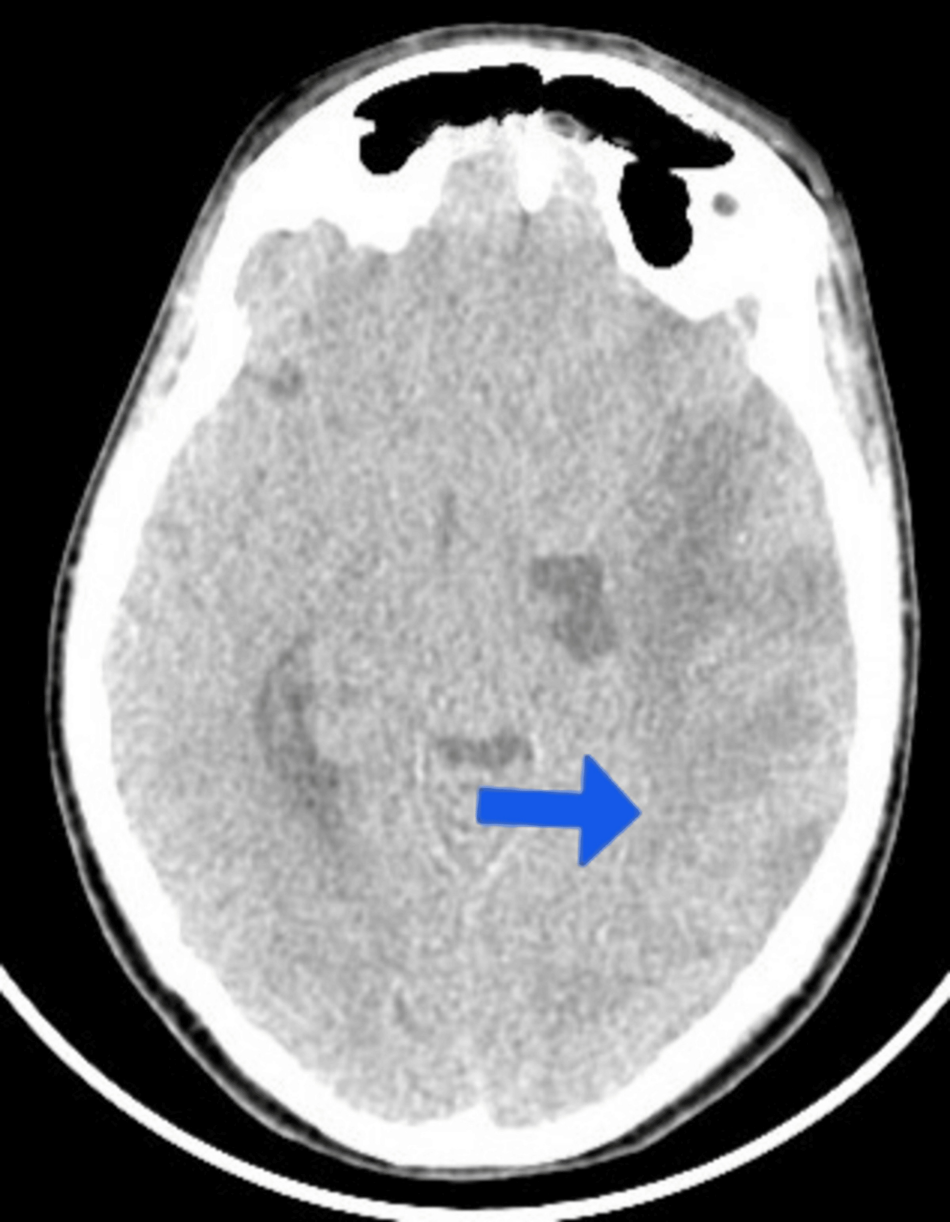
CT brain without contrast-axial view; the blue arrow shows hypodensity with surrounding edema in the left parieto-occipital region

The patient’s condition initially worsened, manifesting as headaches, focal seizures, and altered mental status as quantified by a Glasgow Coma Scale score of 11/15 (E3V4M4). A CT scan was done which showed hypodensities in the left parietal (Figure 1) and occipital lobe (Figure 2) secondary to septic embolus involving the left middle cerebral artery. Neurosurgical consultation advised continued antibiotic management without surgical intervention.

Vancomycin and metronidazole were added to the regimen to increase the coverage of potentially undetected pathogens. The patient showed clinical improvement on the seventh day of treatment. After three weeks of antibiotic injections, the patient was released with supportive care. Following the two-week subsequent follow-up, the patient's recovery had progressed well. After a year of her illness, the patient is currently doing well with no neurological deficits.

## Discussion

André Lemierre initially characterized Lemierre's syndrome in 1936, which is a complex condition that usually follows tonsillitis and includes anaerobic and septic bacterial infections [11]. The illness starts with a peritonsillar infection, progresses to thrombophlebitis involving the internal jugular vein, and can lead to septic embolism of distant organs such as the brain and lungs [12]. The palatine tonsils are the most frequent primary site of infection, accounting for 87.1% of cases [13]. However, other infections, such as those related to dental procedures, mastoiditis, parotitis, sinusitis, otitis, and skin infections, can also precipitate the syndrome [13].

Patients often develop a high fever and chills four to five days after the onset of pharyngitis [14]. The common symptoms include neck discomfort, stiffness, and cervical lymphadenopathy. Swelling and pain around the lower jaw or near the sternocleidomastoid muscle are signs of parapharyngeal involvement, which affects 26-45% of patients [14]. Atypical appearances might arise without fever and a history of pharyngitis. Since the advent of antibiotics, the mortality rate from Lemierre's illness has dropped to as low as 5% [15]. Approximately 10% of affected individuals may have lasting consequences. These include enduring neurological damage due to brain abscesses and meningitis, the need for valve replacement surgery due to endocarditis, and ongoing issues with bone infections known as chronic osteomyelitis [16].

Early and accurate diagnosis is crucial to prevent severe outcomes such as sepsis and death, but it is often delayed owing to the subtle progression of the syndrome and lack of widespread recognition. Imaging techniques such as CT neck with contrast are instrumental in diagnosis, revealing soft tissue edema and the presence of thrombi within the internal jugular vein [14]. The mainstay of treatment for Lemierre syndrome involves intravenous antibiotics that are effective against anaerobic bacteria [15]. Patients typically experience a gradual reduction in fever, with resolution occurring on average within 8-12 days [11].

Although rare, surgical procedures such as ligation and excision of the internal jugular vein might be necessary for situations involving persistent septic emboli drain abscesses or empyema. The use of anticoagulants remains controversial, as there are no randomized studies that support their routine use [14]. 

Advanced Lemierre syndrome poses a serious risk to one's life. According to reports, fatality rates range from 5-18% even with the right medications and treatment. ICU status is typically required for hospital admission, and hospital stays last roughly three weeks on average, as seen in our patient [17]. End-organ damage from septic emboli can cause long-term morbidity in Lemierre syndrome, affecting the kidneys, liver, lungs, and brain. Potential side effects include neurological impairments, long-term respiratory problems, and multi-organ dysfunction, particularly if diagnosis and treatment are postponed [17]. However, with prompt antibiotic treatment, supportive care, and rehabilitation, some patients like the one in our case may fully recover. A favorable prognosis is influenced by early intervention, the lack of major infarctions, and proper rehabilitation.

## Conclusions

In conclusion, the young woman’s case was a clear example of Lemierre’s syndrome triggered by a dental procedure. Despite the initial severity, including fever, neck swelling, dysphagia, and the unexpected discovery of *Eikenella corrodens*, she responded well to tailored antibiotic treatment. The decision to use anticoagulants, although debated, along with the strategic addition of vancomycin, played a crucial role in the patient’s recovery. This case demonstrates the vital need for proper diagnosis, prompt and adequate antibiotic administration, and meticulous evaluation of anticoagulation while managing Lemierre's syndrome. Her progress and recovery at the follow-up appointments without any residual neurological deficits confirmed the effectiveness of the medical intervention and the strength of the patient in overcoming this serious condition. 
